# P68 RNA Helicase (DDX5) Required for the Formation of Various Specific and Mature miRNA Active RISC Complexes

**DOI:** 10.2174/2211536611666220218121640

**Published:** 2022-07-29

**Authors:** Mariette Kokolo, Montse Bach-Elias

**Affiliations:** 1 Instituto de Investigaciones Biomédicas de Barcelona-Consejo Superior de Investigaciones Científicas, Barcelona, Spain

**Keywords:** p68 RNA helicase, miRNAs, mature miRNA active RISC complexes, miR-206, Dicer, Let-7a

## Abstract

**Introduction:**

DEAD-box RNA helicases catalyze the ATP-dependent unwinding of double-stranded RNA. In addition, they are required for protein displacement and remodelling of RNA or RNA/protein complexes. P68 RNA helicase regulates the alternative splicing of the important proto-oncogene *H-Ras*, and numerous studies have shown that p68 RNA helicase is probably involved in miRNA biogenesis, mainly through Drosha and RISC/DICER complexes.

**Objective:**

This study aimed to determine how p68 RNA helicase affects the activity of selected mature miRNAs, including miR-342, miR-330, miR-138 and miR-206, miR-126, and miR-335, and let-7a, which are known to be related to cancer processes.

**Methods:**

The miRNA levels were analyzed in stable HeLa cells containing p68 RNA helicase RNAi induced by doxycycline (DOX). Relevant results were repeated using transient transfection with pSuper/pSuper-p68 RNA helicase RNAi to avoid DOX interference.

**Results:**

Herein, we reported that p68 RNA helicase downregulation increases the accumulation of the mature miRNAs, such as miR-126, let-7a, miR-206, and miR-138. Interestingly, the accumulation of these mature miRNAs does not downregulate their known protein targets, thus suggesting that p68 RNA helicase is required for mature miRNA-active RISC complex activity.

**Conclusion:**

Furthermore, we demonstrated that this requirement is conserved, as drosophila p68 RNA helicase can complete the p68 RNA helicase depleted activity in human cells. Dicer and Drosha proteins are not affected by the downregulation of p68 RNA helicase despite the fact that Dicer is also localized in the nucleus when p68 RNA helicase activity is reduced.

## 1 INTRODUCTION

RNA helicases have been shown to be necessary for RNA decay, ribosome biogenesis, splicing and alternative splicing, mRNA export from the nucleus, and translation initiation [1, 2]. DEAD-box proteins are putative RNA helicases with a characteristic Asp-Glu-Ala-Asp (DEAD) box as one of eight highly conserved sequence motifs involved in ATP hydrolysis [3-5]. P68 RNA helicase is a proliferation-associated nuclear antigen first identified due to its highly specific cross-reaction with the simian virus 40 tumour antigen [3, 4]. Protein expression of p68 and p72 RNA helicases is upregulated in colorectal, prostate, and breast cancer [6-9], thus indicating that helicase activity is key to many cancerous processes [10]. We have previously demonstrated that p68 RNA helicase is an important alternative splicing regulator of the proto-oncogene *c-H-ras* [11-13]. This helicase inhibits the expression of p19 H-Ras [12], a protein that may induce G1/S delay and thus maintain cells in a reversible quiescent state [14]. We previously also showed that p68 RNA helicase regulates survival pathways involving mTOR and MDM2 signals [15].

MiRNAs belong to a class of non-coding RNAs that play an important role in the downregulation of gene expression [16-19]. During miRNA biogenesis, they are initially transcribed as long primary transcripts, known as pri-miRNAs, and are then processed in the nucleus by the RNAse III enzyme Drosha to give pre-miRNAs 60-120 nucleotides that are transported into the cytoplasm [16-19]. These pre-miRNAs are further cleaved by a second RNAse III enzyme (Dicer) to release mature miRNAs with 18-25 nucleotides in length [20, 21]. The mature miRNA duplex is unwound to form the sequence-complex RISC mostly by binding to 3’-untranslated regions (UTRs) of its target mRNA, which represses it at the translational step or induces RNA degradation [20, 21]. Ishizuka *et al.* in 2002 reported that *Drosophila melanogaster* p68 RNA helicase (Dmp68) could be present in the RNA-induced silencing complex (RISC), a sequence-specific complex that mediates RNA interference (RNAi) in Drosophila [22], thus suggesting that p68 RNA helicase could also form part of some sequence-specific RISC complexes. In 2009, Suzuki *et al.* showed that p53 interacts with the Drosha processing complex by associating with the DEAD-box RNA helicase, and these interactions facilitate the processing of primary miRNAs to precursor miRNAs, including miR-16-1, that suppress growth [23].

Herein, we demonstrated that p68 RNA helicase is a necessary component for a subset of sequence-specific RISC complexes, which is required for the final RNA interference (RNAi) of mature miRNAs. Furthermore, we also reported that this required helicase activity is conserved from Drosophila to human cells and that p68 RNA helicase regulates the cellular localization of Dicer.

## 2 MATERIALS AND METHODS

### 2.1 Cultured Cells

HeLa cells were cultured as described previously [14]. Stable HeLa cells containing p68 RNA helicase RNAi induced by doxycycline (DOX) were obtained by co-transfecting pTER-p68 RNA helicase RNAi/pCDNA_6_/TR. The initial selection was performed with zeocin/blasticidin (200/105 μg/ml) and secondary selection by indirect immunofluorescence detection with anti-p68 RNA helicase antibody in cells induced with 2μg/ml DOX. Clones 5, 13, and 17 were maintained at a final zeocin/blasticidin concentration of 100/5 μg/ml, respectively. As DOX interfered in some experiments, we used a range of DOX concentrations (0.2-2μg/ml) to confirm the results obtained. Relevant results were repeated using transient transfection with pSuper/pSuper-p68 RNA helicase RNAi to avoid DOX interference. Additional negative control experiments were performed with the unrelated siRNA B (Santa Cruz Biotechnology, sc-44230) that render similar results as empty pTER and pSuper vectors. We repeated the experiments with pSUPER-scramble (Plasmid #118349 addgene).

### 2.2 Antibodies

Following antibodies were generous gifts: anti-human p68 RNA helicase PAb204 and anti-Dmp68 MAD 1 (both from Dr. F. Fuller-Pace), and anti-HMGA2 CS-2424 (from Dr. Masako Narita). Other antibodies, such as anti-p19 H-Ras was from our research group [11, 14], anti-GAPDH (FL-335) and anti-K-Ras (F234) were obtained from Santa Cruz Biotechnology, anti-ERα (62A3) was obtained by cell signaling, anti-Dicer (H-212) was procured by Santa-Cruz Biotechnology, and anti-Drosha (ab12286) was obtained by ***Abcam***.

### 2.3 Plasmid Construction

Synthesis of pSuper-p68 RNA helicase RNAi has been described previously [12]. PTER-p68 RNA helicase RNAi was obtained in a similar manner using the same sequence as pSuper-p68 RNA helicase RNAi. PsiCheck-HMGA2 3’ UTR, a reporter plasmid containing HMGA2 3’UTR, was a generous gift from Dr. Marcus E. Peter (University of Chicago). PSuper.neo.GFP-pre-miR-206 was obtained by directly cloning the two complementary deoxyoligonucleotides into pSuper.neo.GFP. The expression of mature miR-206 on HeLa cells was examined using the Taqman assays. Dmp68 containing clone was a generous gift from Dr. Akira Ishizuka (University of Molecular Biology, Tokyo), whereas *Drosophila melanogaster* pRK5-Dmp68 and pRK5-Dmp68T were obtained by amplifying the sequences containing specific deoxyoligonucleotides and subsequent cloning in the pRK5 vector. *Drosophila melanogaster* Dmp68T, which contains a deletion of the last thirty-two amino acids, further assures that it remains unaffected by our human p68 RNA helicase RNAi (see also results).

### 2.4 Real-time TaqMan Assays

MiRNAs were extracted using the miRVANA^TM^ miRNA isolation kit, from Ambion Inc. (Austin, Tx) or TRIzol^®^ Reagent (Cat. No.15596-026), from Invitrogen. Isolation was performed according to the manufacturer’s protocol. Mature miRNA real-time TaqMan^®^ assays were performed using Applied Biosystems and repeated three times in three independent experiments.

### 2.5 Quantitative Reverse Transcription-PCR

Stem-loop quantitative reverse transcription-PCR (qRT-PCR) for mature miRNAs was performed using an Applied Biosystem ABI 7000 Real-Time PCR system, as described by the manufacturer. All PCR reactions were run in triplicate, and gene expression, relative to U6, was calculated using the 2-ΔΔCt method [24]. Total H-Ras was detected by performing the assay of Hs00978051_g1 and GAPDH by the assay of HS99999905_m1 housekeeping, both of which were supplied by Applied Biosystems Gene Expression Assays. Assays were run with TaqMan^®^ Universal. cDNA was reverse-transcribed from total RNA samples using Super Scrip III^®^ from Invitrogen. The resulting cDNA was amplified by PCR using TaqMan primers with the TaqMan^®^ Universal PCR Master Mix, No AmpErase^®^ UNG, and analyzed with a 7500 ABI PRISM Sequence Detector System according to the manufacturer’s instructions. MRNA expression was calculated from the relevant signals by normalization with respect to the signal for GAPDH mRNA expression.

### 2.6 Western Blots and Indirect Immunofluorescence

Western blots and indirect immunofluorescence were performed as described previously [11]. Chemiluminescence reaction was detected after 5 min in LAS-4000 Image analyzer (GE Healthcare Life Sciences, Pittsburgh, PA) and quantified using Fujifilm Multi Gaige V3.0 imaging software (GE Healthcare Life Sciences, Pittsburgh, PA).

### 2.7 Rluc/Fluc Assay

This assay was performed using a Dual-Glo™ Luciferase Assay System, according to the manufacturer's instructions (https://www.promega.es/products/luciferase-assays/reporter-assays/dual_glo-luciferase-assay-system/?catNum=E2920#protocols).

### 2.8 Cell Proliferation Assay

HeLa cells were transiently transfected with pSuper.neo.GFP-pre-miR-206 or pSuper.neo.GFP. After three days, cells were collected and plated in 96 wells (500 cells/well) on six microplates in sextuplicate and incubated at 37ºC, 5% CO_2_ with DMEM/10% FBS. After the appropriate time, the cell culture medium was discarded, and the microplates were frozen at -80ºC. Cells were quantified using the green fluorescent dye CyQuant (Invitrogen) according to the manufacturer's instructions. Fluorescence measurements were performed using a microplate reader with excitation at 485 nm and detection at 530 nm.

## 3 RESULTS

### 3.1 P68 RNA Helicase Regulates the Mature miRNA Active RISC Complexes Action of a Specific Set of Mature miRNAs

**Fig. (1):**
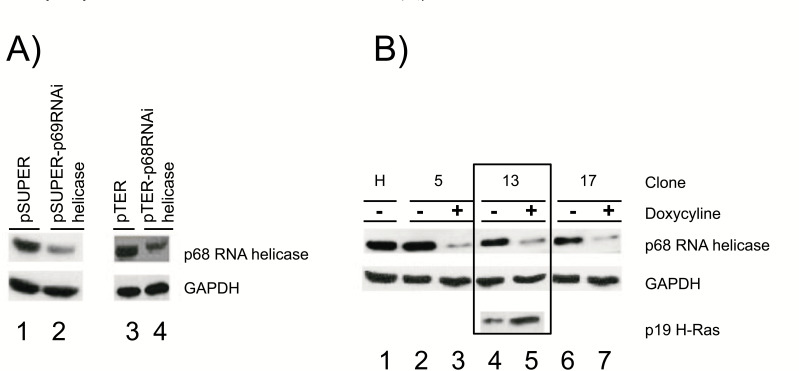
Stable HeLa cells expressing p68 RNA helicase RNAi analyzed by western blots and incubated with anti-p68 RNA helicase, anti-GAPDH, and anti-p19 H-Ras antibodies. **A**) Transient expression with vectors: lane 1: pSuper (negative control); lane 2: pSuper-p68 RNA helicase; lane 3: pTER (negative control); lane 4: pTER-p68 RNA helicase. **B**) Stable and inducible expression of the p68 RNA helicase RNAi: wild-type HeLa control cells (H, lane 1), clones 5 (lanes 2 and 3), 13 (lanes 4 and 5), and 17 (lanes 6 and 7) without (-) or with (+) DOX, 2μg/ml. This figure also shows, with clone 13, how p19 H-Ras increases when p68 RNA helicase is downregulated, as reported previously [12], in lanes 4 and 5. The results were normalized with respect to GAPDH as an internal control. Lane 5 shows an 80% decrease in p68 RNA helicase and a 40% increase in p19 H-Ras. Three independent experiments were performed, each of them per triplicate.

In order to assess how p68 RNA helicase affects the activity of mature miRNAs, we first obtained a stable cell containing an inducible p68 RNA helicase RNAi with the pTER vector. A sequence requirement for this RNAi was previously reported using transient transfections with pSuper vector [12]. Fig. (**1A** and **1B**) show transient and stable/inducible transfections, respectively. Several doxycyclines (DOX)-inducible clones of stable cells were obtained (Fig. **1B**), including clones 5, 13, and 17. Protein extracts from these cells were assayed by western blots of the stated antibodies. As discussed previously, p68 RNA helicase RNAi was found to increase the level of p19 H-Ras, thus indicating that this helicase regulates the alternative splicing of H-Ras (Fig. **1B**) [12]. As doxycycline has previously been found to interfere in some experiments, we evaluate the effect of DOX concentration in each specific assay on wild-type HeLa cells. We mainly used clone 13, as it proved to be more stable during growth, along with a DOX concentration between 0.2-2μg/ml to induce p68 RNA helicase RNAi in this clone, which normally results in 80% p68 RNA helicase downregulation. When DOX was found to interfere, we used transient expressions with pSuper/pSuper p68 RNA helicase RNAi vectors.

We assayed the effect of p68 helicase RNAi on the mature miRNA action of a specific set of miRNAs in both transient and inducible RNAi. This set included miR-342, miR-330, miR-138, and miR-206, which are known to be upregulated by p19 H-Ras [25]. Furthermore, we also selected miR-126 and miR-335, which significantly reduced the ability of CN34-LM1 and CN34-BoM1 cells to metastasize to the lung [26-28] and let-7a, which is known to target Ras genes [29, 30]. Fig. (**2A**, **B**) shows that the downregulation of p68 RNA helicase induces the upregulation of miR-126, let-7a, miR-206, and miR-138, whereas miR-335 and miR-330 remain almost unaffected and miR-342 is found to be downregulated. Similar results were obtained during transient expression. DOX was found to interfere with miR-206, miR-138, miR-335, miR-342, and miR-330 expressions; therefore, transient expression in wild-type HeLa cells was the method of choice in these cases.

### 3.2 Many Protein Targets of the Upregulated miRNAs Remain Unaffected

We then examined the known protein targets of the miRNAs that were found to be upregulated upon p68 RNA helicase interference, namely HMGA2, K-Ras, and H-RAs for let-7a [29, 31] and ERα for miR-206 [32] by western blot. We also assayed DICER for miR-330 (putative target for miR-330 as obtained by TARGETSCAN), a miRNA that remained unaffected in this interference assay. Let-7a also has a Dicer exon as a target [33], as shown in Fig. (**3**); this Dicer protein remains unaffected. We, therefore, concluded that although a clear upregulation of mature let-7a and miR-206 was observed, these two miRNAs were not able to downregulate their known targets, thus indicating that p68 RNA helicase could also be required for duplex unwinding RISC formation of these two miRNAs and that they may accumulate as they are not further used in the mature miRNA active RISC complex.

### 3.3 MiR-206 Downregulates ERα, p68 RNA Helicase, and HMGA2

We decided to further evaluate the action of miR-206 for the following reasons: a) it has a low expression in HeLa cells, and it is the one that presents a greater fold change, as shown in Fig. (**2**), b) it targets the important protein ERα, and c) it plays a crucial role in lung cancer [34]. Fig. (**4A**) depicts that overexpression of pre-miR-206 decreases HeLa cell growth, which is an additional miR-206 property that makes it more attractive to study its complete function. This cell arrest has been previously reported by Song *et al*. [35]. We have found that HMGA2 and p68 RNA helicase 3’-UTRs contain putative sites for miR-206 binding (putative targets for miR-206 as obtained by TARGETSCAN). To test this possibility, we obtained a pSuper vector containing pre-miR-206 and transiently transfected with wild-type HeLa cells. As shown in Fig. (**4B**), miR-206 induces downregulation of ERα (a known target 3’-UTR), p68 RNA helicase, and HMGA2 (lanes 1). We also checked a psiCheck-HMGA2-3’UTR reporter vector containing the 3’-UTR of HMGA2. As shown in Fig. (**4C**), pre-miR-206 interferes with the 3’-UTR of HMGA2 (column 2).

### 3.4 P68 RNA Helicase from *Drosophila melanogaster* Complements the Human Downregulated p68 RNA Helicase and Increases Interference of Upregulated miRNAs

We chose p68 RNA helicase from *Drosophila melanogaster* (Dmp68) to complement clone 13 plus DOX. Thus, we observed that the human RNA target for the RNAi sequence in clone 13 is quite different from Dmp68. We obtained the pRK5-Dmp68 human expression vector along with a second pRK5-Dmp68T vector containing a C-terminal truncated form of Dmp68 (deletion of the last thirty-two amino acids). We found that the Dmp68T form was more active in HeLa cell complementation assays (not shown); therefore, we used both Dmp68 and Dmp68T in specific experiments. Fig. (**5A**) shows that Dmp68 complements the depleted human p68 RNA helicase activity and induces twice as much downregulation of ERα (lane 1). Similar results were obtained with transient expressions with pSuper vectors. Dmp68 was co-transfected with psiCheck-HMGA2-3’UTR vector in clone 13 and human p68 helicase RNAi induced with DOX. Only Dmp68 without DOX showed effect by itself on luciferase activity, as shown in Fig. (**5B**) column 1. Dmp68T complements the human p68 downregulation and increases (twice) the interference of the HMGA2 3’-UTR (compare columns 2 and 3). Dmp68 also rescues p68 RNA helicase activity to a lesser extent (compare columns 3 and 4 with column 2). We, therefore, concluded that p68 RNA helicase is necessary for the action of mature let-7a and miR-206 in the cytoplasm, probably as a result of a specific unwinding activity and that higher amounts pf 68 RNA helicase might increase the HMGA2 RNA interference.

### 3.5 P68 RNA Helicase RNAi Increases the Import of Dicer to the Nucleus

To further understand the role of p68 RNA helicase in the biogenesis and activity of miRNAs, we determined whether the RNAi of p68 RNA helicase affects any of the key proteins required for miRNA biogenesis. As already shown in Fig. (**3**), Dicer did not affect by the RNAi of the p68 RNA helicase, and a similar situation was found for Drosha (Fig. **6A**). However, we observed that p68 RNA helicase RNAi induced a partial import of Dicer granules to the nucleus, as shown in Fig. (**6B**) (lane 1, 15-20% of total Dicer), thus indicating that p68 RNA helicase RNAi affected both the unwinding activities of mature miRNA active RISC complexes and the amount of Dicer available in the cytoplasm.

## 4 DISCUSSION

**Fig. (4):**
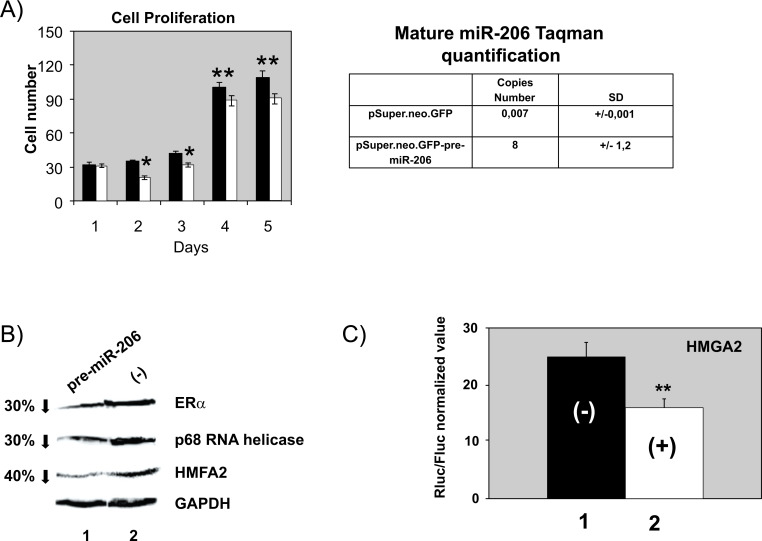
MiR-206 overexpression regulates ERα, p68 RNA helicase, and HMGA2. **A**) On the left, it shows how the overexpression of the pSuper.neo.GFP-pre-miR-206 (white columns) decreases cell growth in HeLa cells, (*) *P*=0.01 and (**) *P*=0.05 (Student’s *t*-test). On the right, the table shows the Taqman quantification of the overexpression of mature miR-206. **B**) Western blots, incubated with anti-ERα, anti-p68 RNA helicase, anti-HMGA2, and anti-GAPDH, with protein extracts from wild-type HeLa cells transiently co-transfected with pSuper.neo.GFP-pre-miR-206 vector (lane 1) and pSuper.neo.GFP empty vector (lane 2). **C**) Wild-type HeLa cells transiently transfected with psiCHECK-HMGA2 reporter vector containing the HMGA2 3’-UTR. Lane 1: wild-type HeLa cells transiently transfected with pSuper.neo.GFP; and lane 2: with pSuper.neo.GFP-pre-miR-206 vector. MiR-206 action, normalized by the Rluc/Fluc detection assay, shows a 1.7-fold decrease in HMGA2 3’-UTR (in column 2, *P*<0.01, Student’s *t*-test). Three independent experiments were performed, each of them per triplicate.

**Fig. (5):**
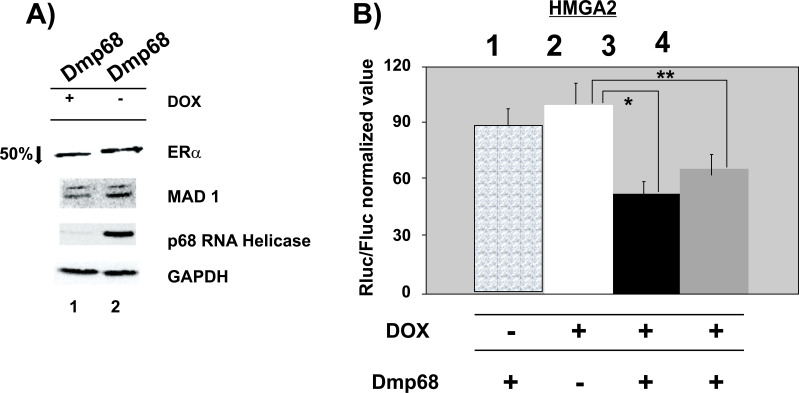
The expression of Dmp68 in clone 13 reduced the ERα level when human p68 RNA helicase was depleted. **A**) Clone 13 with (lane 1) and without (lane 2) DOX. pRK5-Dmp68 vector was expressed in both lanes. Western blots were incubated with anti-ERα, anti-MAD1 (anti-Dmp68), anti-p68 RNA helicase, and anti-GAPDH. Differences in MAD1 intensities were due to the transient transfection levels and not the RNAi present in the cell. Similar results were obtained with transient transfections where lane 1 contained higher amounts of MAD1. The decrease of ERα in lane 1 was 50%. **B**) PRK5-Dmp68 and psiCHECK-HMGA2 vectors were co-transfected in clone 13 in the presence or absence of DOX. Column 1: pRK5-Dmp68T without DOX; column 2: pRK5 empty vector with DOX; column 3: pRK5-Dmp68T with DOX; column 4: pRK5-Dmp68 with DOX. Dmp68 action, normalized by the Rluc/Fluc detection assay, shows a 1.9- and 1.5-fold decrease in HMGA2 3’-UTR level (columns 3 and 4, respectively). (*) *P*<0.001 and (**) *P*<0.01 (Student’s *t*-test). Three independent experiments were performed, each of them per triplicate.

**Fig. (6):**
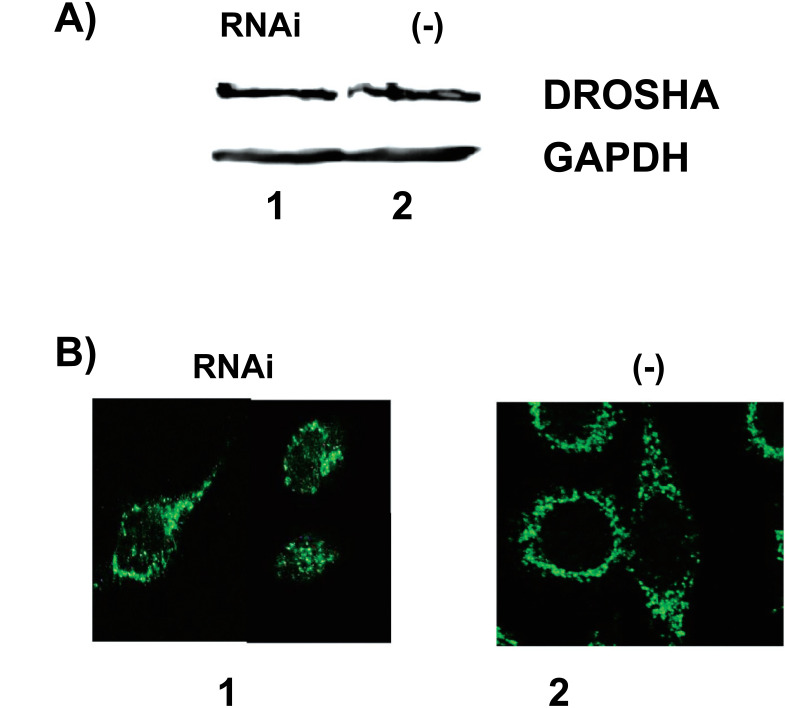
P68 RNA helicase RNAi induces partial nuclear import of Dicer. **A**) A western blot with anti-Drosha and anti-GAPDH. HeLa cells transiently transfected with pSuper-p68 RNA helicase RNAi (lane 1) and negative control pSuper empty vector (lane 2). **B**) Indirect immunofluorescent detection of Dicer in HeLa cells transiently transfected with pSuper-p68 RNA helicase RNAi (lane 1) or pSuper empty vector (lane 2). Quantification of immunofluorescence in nuclei versus cytoplasm indicates that 15-20% of the total Dicer is in nuclei containing p68 RNA helicase RNAi. Three independent experiments were performed, each of them per triplicate. *Bar,* 8 μm.

Ishizuda *et al*. reported the isolation of RISC complexes containing FMR1, AGO2, and Dmp68 from Drosophila in 2002 [22]. They showed that when Dmp68 expression was repressed, EGFP silencing by RNAi was completely abolished, thus indicating that Dmp68 plays an essential role in the RNAi pathway [22]. Our results agree with the previously reported findings, involving Drosophila; thus, when human p68 RNA helicase is downregulated, there is an accumulation of a set of miRNAs that cannot silence their protein targets. The Drosha complexes are rather heterogeneous and can be divided into a) Drosha/DGCR8, b) Drosha/DGCR8 plus p68 and p72 RNA helicases, c) Drosha/DGCR8 plus hnRNP A1, d) Drosha/DGCR8 plus SMAD/p68 RNA helicase, and e) Drosha in splicing complexes containing miRtrons [20]. Here, we discovered a new role for p68 RNA helicase as a specific factor required for the action of a subset of mature miRNAs. We paid particular attention to let-7a and miR-206, as let-7a is an important marker in many cancers and also targets H-Ras [29], and miR-206 is already known to be upregulated by proto-oncogene p19 H-Ras [25]. These results were in agreement with previous findings of studies conducted by Suzuki *et al*. and Salzman *et al*. In these studies reported that p68 RNA helicase in the nucleus interacts with the Drosha-containing microprocessor complex and promotes the processing of a subset of pri-mRNAs [23, 36] and p68 RNA helicase also facilitates the loading of let-7 to miRISC by the action of its unwinding activity on the let-7 miRNA precursor complex in the cytoplasm [36]. Therefore, our experiments mainly addressed ERα interference by miR-206 and HMGA2 interference by let-7a. We further demonstrated that miR-206 could also target HMGA2 and p68 RNA helicase mRNAs (Fig. **4**). Additionally, we demonstrated that miR-206 overexpression reduces HeLa cell growth (Fig. **4A**). It was previously reported that p68 RNA helicase is required for let-7 miRNA to function in HeLa cells [36] with a let-7 responsive reporter vector [*lin41(s) WT*], which agrees with the results presented in this study. Herein, we further reported that p68 RNA helicase RNAi induces an increased concentration of mature let-7a miRNA, as shown in Fig. (**2**), but not the downregulation of its protein targets (HMGA2, K-Ras, H-Ras, or Dicer), as shown in Fig. (**3**). This finding suggests that p68 RNA helicase is also needed to form the mature miRNA-active RISC complex, as previously reported [36]. To validate this, we complemented cells containing depleted human p68 RNA helicase with Dmp68. As the C-terminal region contains several putative binding sites for human RNAi and is also the less conserved region [22], we also complemented it with a C-terminal truncated Dmp68T, which was subsequently found to be more active (Fig. **5**). Our results showed that Dmp68 and Dmp68T induce the reduction of HMGA 3’-UTR, as shown in Fig. (**5B**), in cells containing human p68 RNA helicase RNAi. This finding also indicates the conservation of this RISC mechanism from Drosophila to human cells. A similar effect was seen with the miR-206 protein target; when complementing with Dmp68, the downregulation of this ERα was induced, as seen in Fig. (**5A**). However, we found, so far, no secondary structure similarities between let-7a and miR-206 and either pre-miRNAs or mature miRNAs that could explain the action of p68 RNA helicase. Nevertheless, it is possible that p68 RNA helicase could be necessary to unwind some specific secondary structures on the target 3’-UTR in order for them to be accessible to the miRNA.

This study has also identified miRNAs that do not change their level after p68 RNA helicase RNAi downregulation, specifically miR-335 and miR-330, and one (miR-342) that decreases its level. However, we cannot rule out that these miRNAs also require p68 RNA helicase to form the mature miRNA-active RISC complexes containing mature miRNAs. It was found that several other miRNAs also depend on p68 RNA helicase in the Drosha complex to process pri-miRNAs. When we observe mature miRNAs, we may simply be detecting the two major aspects, namely less pre-miRNA formation due to less Drosha complex formation and accumulation of inactive mature miRNAs. This could indicate that helicase should also be necessary for the formation of the mature miRNA-active RISC complexes of the miR-335, miR-330, and miR-342.

Interestingly, p68 RNA helicase RNAi did not affect Dicer, Drosha, as seen in Figs. (**3** and **6**), and Argonaute (our unpublished results) protein levels, although we observed a greater number of Dicer granules in the nucleus (15-20% of total Dicer) when p68 RNA helicase was downregulated, thus indicating that unwinding activity is also necessary for the localization of Dicer. Emmerth *et al.* showed that nuclear retention of fission yeast Dicer is a prerequisite for RNAi-mediated heterochromatin assembly [37]. These authors analyzed the subcellular localization of Dcr1 (Dicer *Schizosaccharomyces pombe*) and found that the nuclear accumulation of Dcr1 depends on a short motif that impedes nuclear export promoted by the double-stranded RNA binding domain of Dcr1 [37]. The absence of this motif renders Dcr1 mainly cytoplasmic. They also suggested that Dicer proteins are shuttling proteins [37]. Herein, we observed that such shuttling would depend on the level of p68 RNA helicase activity, as shown in Fig. (**6**), where p68 RNA helicase RNAi induces an increasing amount of human Dicer in the nucleus. Furthermore, Dcr1 has an extended C terminus whereas human Dicer does not, which can explain why human Dicer localizes mainly in the cytoplasm [37]. As a double-stranded RNA binding domain of Dcr1 is chiefly responsible for the nuclear accumulation [37], we suggest that in the case of human Dicer, double-stranded RNA domains could be activated in the absence of helicases, thereby allowing the nuclear import. Later reports showed that Dicer is a shuttling protein with a non-classical nuclear localization signal, which localizes in cytoplasm or nuclei according to steady-state conditions [38-41].

## CONCLUSION

In summary, p68 RNA helicase is necessary for mature miRNA-active RISC complex action of certain mature miRNAs. Furthermore, it regulates at least two important miRNAs, namely let-7a and miR-206, which are closely involved in cancer and metastatic processes.

## Figures and Tables

**Fig. (2):**
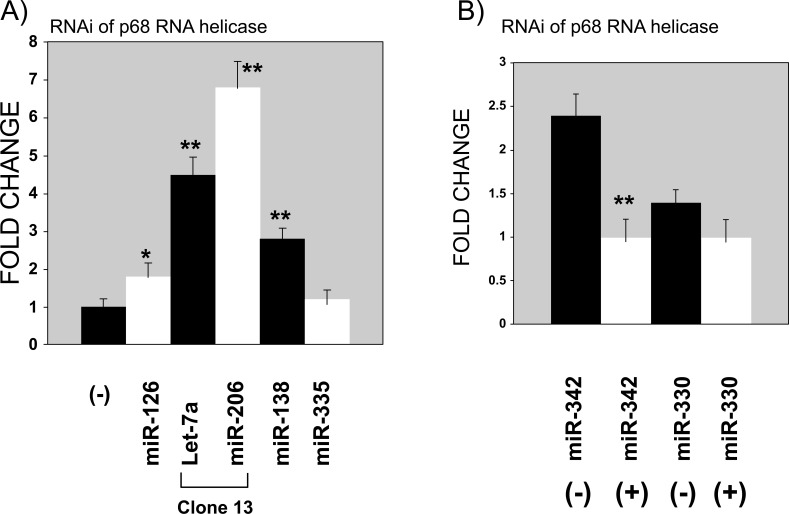
Mature miRNA expression is affected by p68 RNA helicase RNAi. **A**) Real-time Taqman quantifies mature miRNAs when p68 RNA helicase RNAi is expressed. Column (-) is the control set to 1 of transiently expressed empty vector pSuper or clone 13 without DOX. Other columns were normalized to this negative control in each independent experiment. DOX does not interfere with miR-126 or let-7a in wild-type HeLa cells, therefore clone 13 without (column “-”) and with DOX (columns miR-126 and let-7a) was chosen to quantify these miRNAs. Both miRNAs were also checked by transient expression with the pSuper/pSuper-p68 RNA helicase RNAi with similar results. DOX was found to interfere with the expression of miR-206, miR-138, and miR-335; therefore transient expressions were performed with the pSuper/pSuper-p68 RNA helicase vectors in this case. **B**) Real-time Taqman quantifies mature miRNAs when p68 RNA helicase RNAi is expressed. Column (-) is the control set to 1 of transiently expressed empty vector pSuper. Other columns were normalized to this negative control in each independent experiment. DOX interferes with the expression of miR-342 and miR-330; therefore, transient expressions were performed with the pSuper/pSuper-p68 RNA helicase vectors in this case. The results were normalized with respect to snRNA U6 as an internal control. The unrelated miRNA siRNA B was also used as negative with similar results. Three independent experiments were performed, each of them per triplicate. (**) *P*<0.01 and (*) *P*< 0.05 (Student’s *t*-test).

**Fig. (3):**
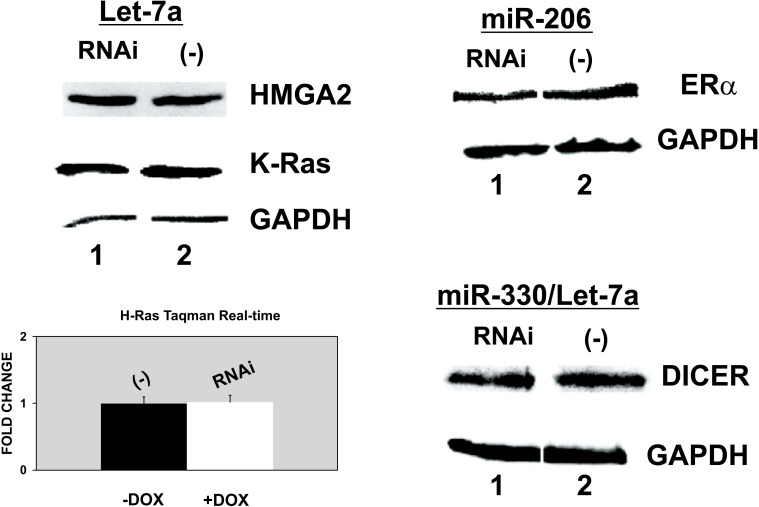
High expression of let-7a and miR-206 obtained by p68 RNA helicase RNAi did not affect target protein levels. **Left:** Clone 13 with (lane 1) and without (lane 2) DOX. Let-7a targets: HMGA2, K-Ras (by western blot) and H-Ras (assayed by Taqman Real-time cDNA quantification below). **Right:** Transient expression with pSuper-p68 RNA helicase RNAi (lane 1) and pSuper (lane 2). Western blot incubated with anti-ERα(target for miR-206) and anti-DICER (target for miR-330 and let-7a). The results were normalized with respect to GAPDH as an internal control. No protein showed any change. Three independent experiments were done, each of them per triplicate.

## Data Availability

Not applicable.
